# Solitary Fibrous Tumors: Clinicopathologic Spectrum and Risk Stratification From Tertiary Care Centers in India

**DOI:** 10.7759/cureus.117080

**Published:** 2026-09-28

**Authors:** Rakhi Jagdale, Jaydeep N Pol, Anand Bhosale, Ajay L Sabnis, Heena Shah, Girish Anandrao Kadkol, Abhijit S Pethkar

**Affiliations:** 1 Pathology, Shri Siddhivinayak Ganapati Cancer Hospital, Miraj, IND; 2 Pathology, Mahatma Gandhi Cancer Hospital, Miraj, IND; 3 Pathology, DY Patil University School of Medicine and Pushpalata DY Patil Hospital, Pune, IND; 4 Oncopathology, Mahatma Gandhi Cancer Hospital, Miraj, IND; 5 Pathology, Dr. N.Y. Tasgaonkar Institute of Medical Sciences, Karjat, IND; 6 Surgical Pathology, Mahatma Gandhi Cancer Hospital, Miraj, IND

**Keywords:** extra-pleural, hemangiopericytoma, malignant transformation, nab2-stat6 fusion, solitary fibrous tumor

## Abstract

Solitary fibrous tumor (SFT) is an uncommon fibroblastic mesenchymal neoplasm that may arise at virtually any anatomical site, but is more common in the pleural location. SFTs are characterized by a prominent, branching, thin-walled, dilated (staghorn) vasculature and NAB2-STAT6 gene rearrangement. Although most SFTs pursue an indolent clinical course, a subset demonstrates aggressive behavior with local recurrence, distant metastasis, or malignant transformation.

The primary aim of the study was to study the clinicopathological profile and risk stratification profile of SFT with attention to atypical sites. The secondary aim was to study the Immunohistochemical profile and malignant transformation frequency in a series of SFTs in a defined population. This is a retrospective study that included 20 cases of SFT diagnosed over a defined period of time. Of these 20 cases, six (30%) are pleural and 14 (70%) are extra-pleural. Of the extra-pleural locations, some rare locations were noted in the temporal lobe and para-testicular region. Histologically, most tumors demonstrated the characteristic patternless architecture with alternating hypercellular and hypocellular areas, branching ("staghorn") vasculature, and variable collagen deposition. Immunohistochemistry was performed and all the cases showed strong positivity for vimentin, CD34 and STAT6. Malignant transformation was seen in two cases to fibrosarcoma and one case showed dedifferentiation to angiosarcoma.

We also did risk assessment of the cases and found that out of 20 cases there were 17 cases in the low-risk category, two cases in the intermediate category and one case in the high-risk category. Long-term follow-up was not available.

To conclude, SFT exhibits a broad morphological spectrum with variable biological behavior. Comprehensive histopathological evaluation combined with STAT6 immunohistochemistry and the Demicco risk assessment model provides a useful framework for prognostic stratification.

## Introduction

Solitary fibrous tumor (SFT) is a rare tumor arising in the fibroblastic tissues of various anatomical sites. SFTs are proliferations that are extremely varied with a wide range of histomorphologic patterns posing a diagnostic challenge [1]. Clinically, they present as slow-growing, painless masses producing localized symptoms, such as chest pain and breathlessness in pleural tumors; abdominal distention and constipation in abdominopelvic tumors; and nasal obstruction and voice change in head and neck tumors.

SFTs affect men and women equally, with peak incidence between 20 and 70 years [2]. SFTs are well-circumscribed masses commonly measuring 1-25 cm (median 5-8 cm) [3]. Grossly, the tumors are nodular, tan to reddish brown, occasionally showing hemorrhage and cystic degeneration. Microscopically, they are composed of haphazardly arranged spindled to ovoid cells with indistinct pale eosinophilic cytoplasm within a variably collagenous stroma. There are admixed hyalinized, staghorn-shaped blood vessels. The histological spectrum is wide, ranging from pauci-cellular lesions with abundant stromal keloidal-type collagen to extremely cellular tumors with closely packed cells with little intervening stroma. Commonly, they are characterized by low mitotic counts, without significant pleomorphism or necrosis. Malignant transformation in SFTs is characterized by increased cellularity, marked nuclear atypia, a high mitotic index (>four mitoses per 10 high-power fields (HPF)), and the presence of necrosis. [4]

NAB2-STAT6 gene fusions are pathognomonic for SFT [5]. Detection of the fusions is difficult by conventional cytogenetic methods owing to their proximity to chromosome 12q. STAT6 immunohistochemistry is a sensitive and specific surrogate for all fusions.

SFTs are rare mesenchymal neoplasms first documented and frequently encountered in the pleura [1]; however, in this study, we present cases occurring in unusual sites, namely lung/mediastinum, rib, supraclavicular and infraclavicular region, temporal region, orbit, thigh, cervical region, pouch of Douglas, paratesticular region and lumbar region. The primary aim of the study was to study the clinicopathological profile and risk stratification profile of SFT with attention to atypical sites. The secondary aim was to study the Immunohistochemical profile and malignant transformation frequency in a series of SFTs in a defined population.

## Materials and methods

This is a retrospective observational study performed in joint collaboration by Shri Siddhivinayak Ganpati Cancer Hospital and Mahatma Gandhi Cancer Hospital, Miraj, from the period of 2011 to 2022. The ethical clearance for the study was obtained (IEC no - ERC/1245/Inst/MH/2019/RR-24). The clearance was taken from the ethical committee of Saishwari Clinic for Mental Health as the ethical committees of both Shri Siddhivinayak Ganpati Cancer Hospital and Mahatma Gandhi Cancer Hospital were temporarily not functional, so due to emergency reasons the clearance was obtained from another neutral committee. The laboratory records were searched for cases diagnosed as hemangiopericytoma/SFT during the given period. The cases were consecutive. A total of 26 cases were studied. The medical records of all patients were reviewed retrospectively for clinical presentation, operative procedure performed, and histopathological diagnosis for data collection.

The inclusion criteria included all cases of SFT with adequate clinical data and confirmed by immunohistochemistry (IHC). Duplicate cases of biopsy and resection specimens of the same patients were prevented by entering the data in an Excel sheet (Microsoft, Redmond, WA, USA) in alphabetical order. Cases with inadequate tissue, incomplete clinical details, or poor-quality histological sections precluding definitive evaluation were excluded. Out of 26 cases, six cases were excluded.

Clinical information was obtained from pathology requisition forms and hospital medical records. The following parameters were recorded: age, sex, anatomical site, clinical presentation, radiological findings (where available), surgical procedure performed and follow-up data including recurrence, metastasis, and survival (where available).

Tissue specimens from trucut/excisional biopsies were fixed in 10% neutral-buffered formalin. Gross examination included assessment of tumor size, consistency, cut surface, and areas of hemorrhage and necrosis. Representative sections were processed, embedded in paraffin, and stained with hematoxylin and eosin for microscopic evaluation of the SFT. 

IHC was carried out using an automated antigen retrieval system (BioGenex EZ-Retriever system V.3; BioGenex, Fremont, CA, USA) and an automated IHC staining system (BioGenex i6000 Diagnostics system). The antibodies used in this series were ready-to-use and undiluted. All the antibodies were from BioGenex except STAT6 which was from Cell Marque (Rocklin, CA, USA). The nuclear staining for STAT6 was considered diagnostic when diffuse (70%) and strong. CD34, BCL-2, and CD99 expressions were assessed as positive or negative based on strong and diffuse (>70%) membranous/cytoplasmic staining patterns. The Ki-67 labeling index was estimated as the percentage of positively stained tumor nuclei in areas of highest proliferative activity and the index was manually calculated by counting across approximately 10 HPFs. 

Histological and IHC were reviewed to confirm the diagnosis of an SFT by four senior pathologists with more than 20 years of experience. Any discrepancies were resolved by consensus. The cases were classified according to the WHO 2022 classification of soft tissue tumors. The essential criteria used were a histologically spindle tumor with stag-horn vasculature and positivity for STAT6. The microscopic field area was 1.96 mm2.

The slides were reviewed and histomorphological features assessed were tumor size (maximum dimension), cellularity, nuclear pleomorphism, mitotic count (per 10 HPF/1.96 mm2), tumor necrosis (area of necrotic tissue/total tumor x 100), hemorrhage, dedifferentiation or malignant transformation and lymphovascular invasion (if present).

Malignant transformation was diagnosed based on established histological criteria, including marked hypercellularity, moderate to severe nuclear atypia, mitotic activity of ≥4 mitoses per 10 HPF, tumor necrosis, infiltrative growth, and/or dedifferentiated morphology. Any heterologous differentiation, including angiosarcomatous transformation and fibrosarcomatous transformation, was documented. 

All tumors were stratified according to the modified Demicco risk assessment model [6], incorporating patient age, tumor size, mitotic count and tumor necrosis.

Patient age was scored as 0 if < 55 years and 1 if ≥55 years. Mitotic activity was scored as 0 if <1 mitotic figure/10 HPF, 1 if 1-3 mitotic figures/10 HPF, or 2 if ≥4/10 HPF. Tumor size was scored as 0 if < 5cm, 1 if 5 to < 10cm, 2 if 10 to < 15cm, or 3 if ≥ 15cm. Necrosis was scored 0 if <10% and 1 ≥ 10%.

Based on the cumulative score, tumors were classified into low (0-3), intermediate (4-5), or high-risk (6-7) categories for metastatic potential/aggressive behaviour [6].

## Results

In this study, we present 20 cases diagnosed as SFTs at various sites. There were nine males and 11 females (Figure 1). The mean age at diagnosis was 50.9 years (SD 12.7 years) (Table 1). There were six cases of SFT in the pleura, one in the lung mediastinum, two cases in the orbit, one in the supraclavicular region, one in the infraclavicular region, one in the cervical region, one in the rib, one in the inguinal region, one in the para-testicular region, one in the lumbar region, two cases were in the thigh, one presented in the pouch of Douglas and another one presented as a temporal space-occupying lesion (Table 2).

**Figure 1:**
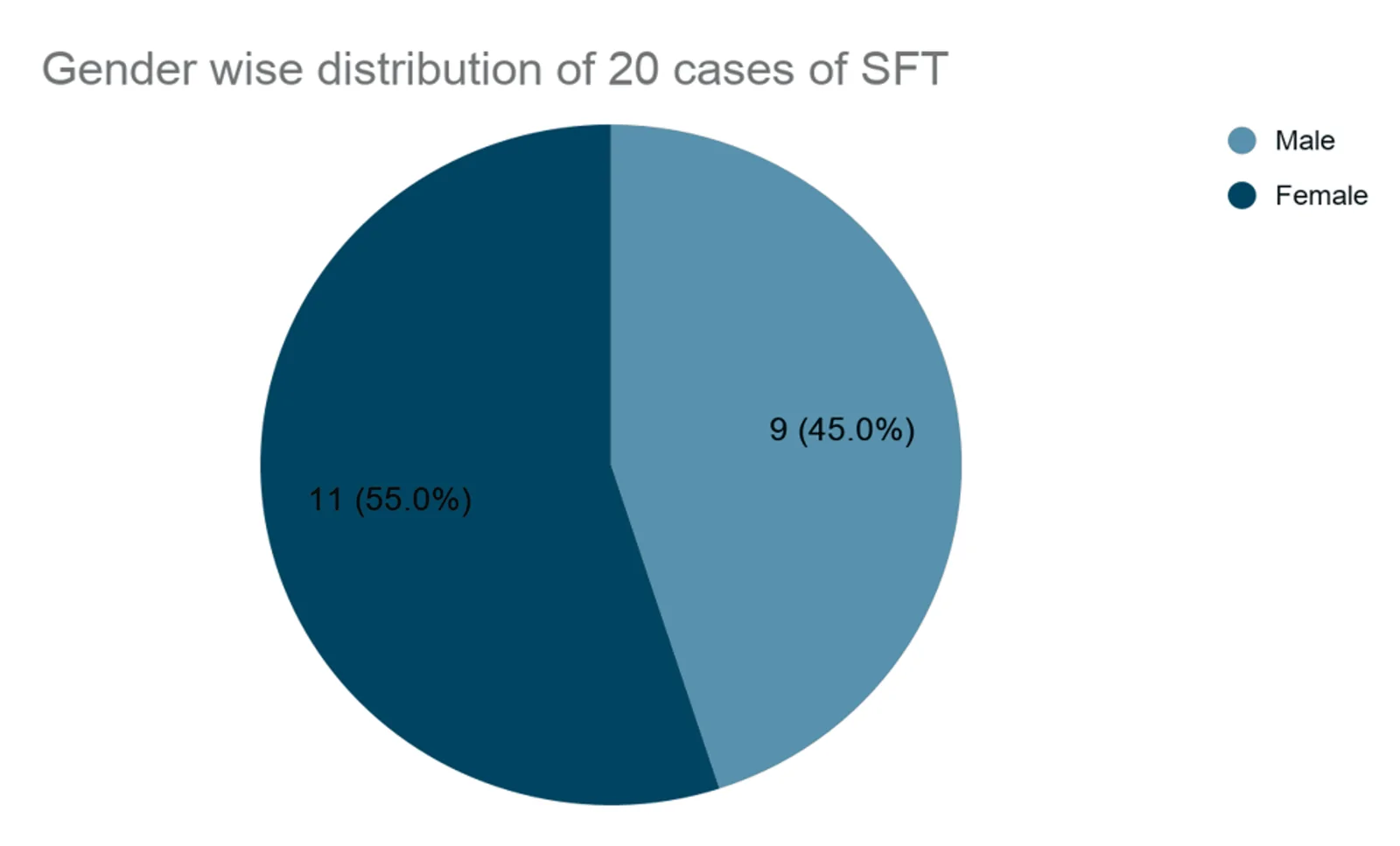
Pie chart showing gender distribution. The pie chart shows the gender distribution of cases, with a slight female preponderance SFT = solitary fibrous tumor

**Table 1:** Age-wise distribution of cases of solitary fibrous tumor (SFT).

Age group	No of patients
10-20	0
21-30	2
31-40	3
41-50	4
51-60	7
61-70	3
71-80	1
Total	20

**Table 2:** Location of tumors.

Location of tumor		No of cases	Total no of cases
Pleural/intra thoracic	Pleura	6	8
	Lung/mediastinum	1	
	Rib	1	
Head and neck	Left supraclavicular region	1	6
	Left infraclavicular region	1	
	Right temporal region	1	
	Orbit	2	
	Cervical region	1	
Extremity	Thigh	2	3
	Inguinal region	1	
Intra-abdominal/ pelvic	Pouch of Douglas	1	3
	Paratesticular region	1	
	Lumbar region	1	

Most tumors were at least moderately cellular with staghorn vasculature and mitosis ranging from 0-5/10 HPF. IHC was carried out in all cases. All 20 cases were positive for CD34, STAT6 and vimentin; 12 cases were positive for BCL2; and 10 cases were positive for CD99 (Figure 2).

**Figure 2:**
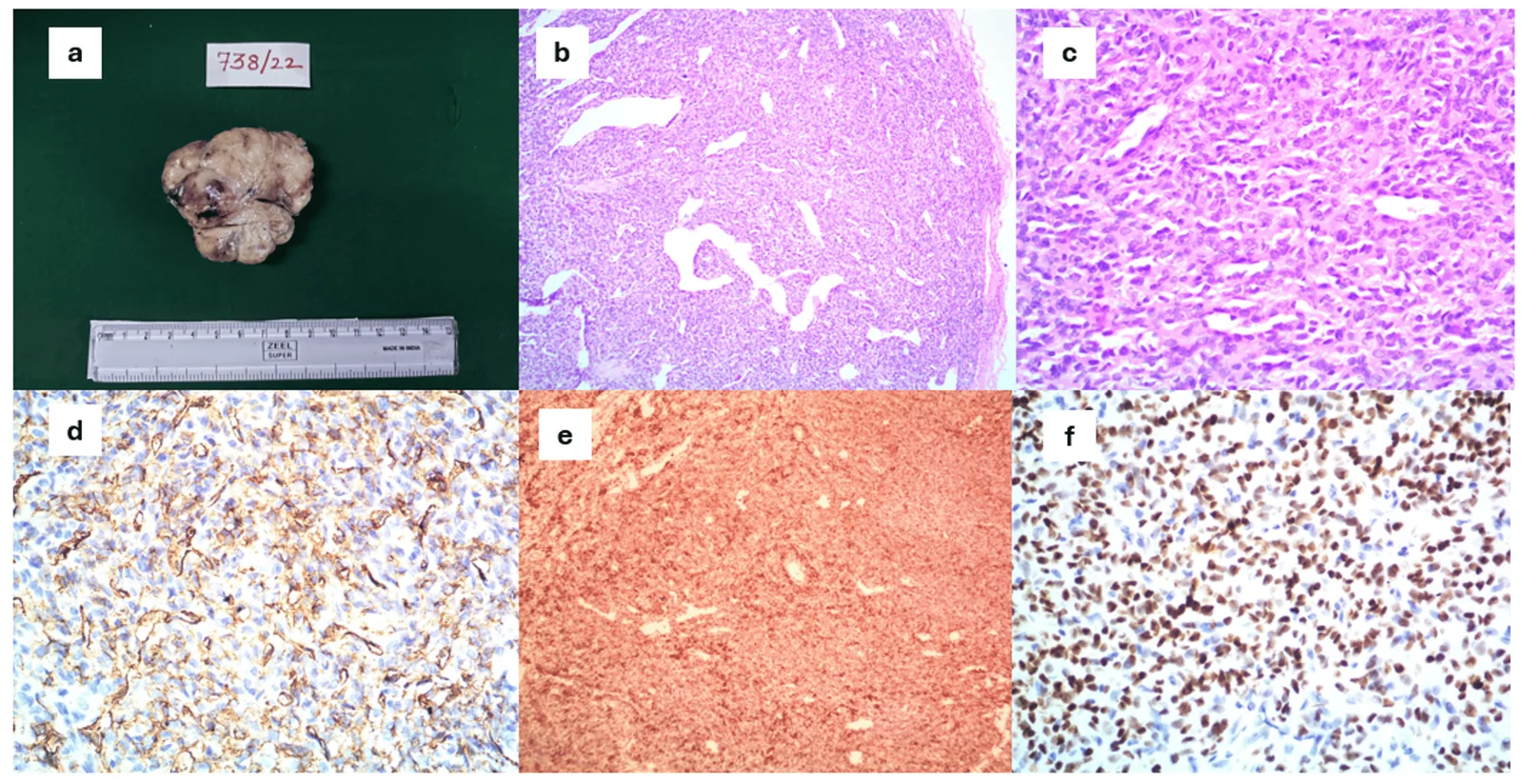
Pleural solitary fibrous tumor (SFT) gross, microscopic and immunohistochemistry (IHC) images. (a) Gross photo of a pleural SFT showing well-circumscribed lobulated tumor with grey-white areas; (b & c) H&E images showing ovoid to spindle cells arranged in storiform pattern with staghorn (hemangiopericytoma-like) vasculature (x100, x400); (d) IHC showing cytoplasmic positivity for CD34 (x100) (e) IHC showing cytoplasmic dot like positivity for CD99 (x200); (f) IHC showing strong nuclear positivity for STAT6 (x400)

In our study, three cases showed features associated with aggressive behavior, including increased mitotic activity (>4/10 HPF), nuclear atypia, and focal necrosis. These lesions include one case each involving the orbit (malignant transformation), the para-testicular region (fibrosarcomatous dedifferentiation) and the posterior thigh (angiosarcomatous dedifferentiation).

The case from the orbit was a 57-year-old male who presented with proptosis and lagophthalmos of the left eye. This patient was an already operated case of SFT eight years back and was disease-free. Now he had the present complaints. The CT scan showed a retrobulbar mass in the left eye. The H&E showed a spindle cell tumor with typical SFT morphology but with marked nuclear atypia and necrosis. IHC showed positivity for STAT6, vimentin, CD34 and BCL2 with a Ki-67 labelling index of 20% while it was negative for CD99 (Figures 3-5).

**Figure 3:**
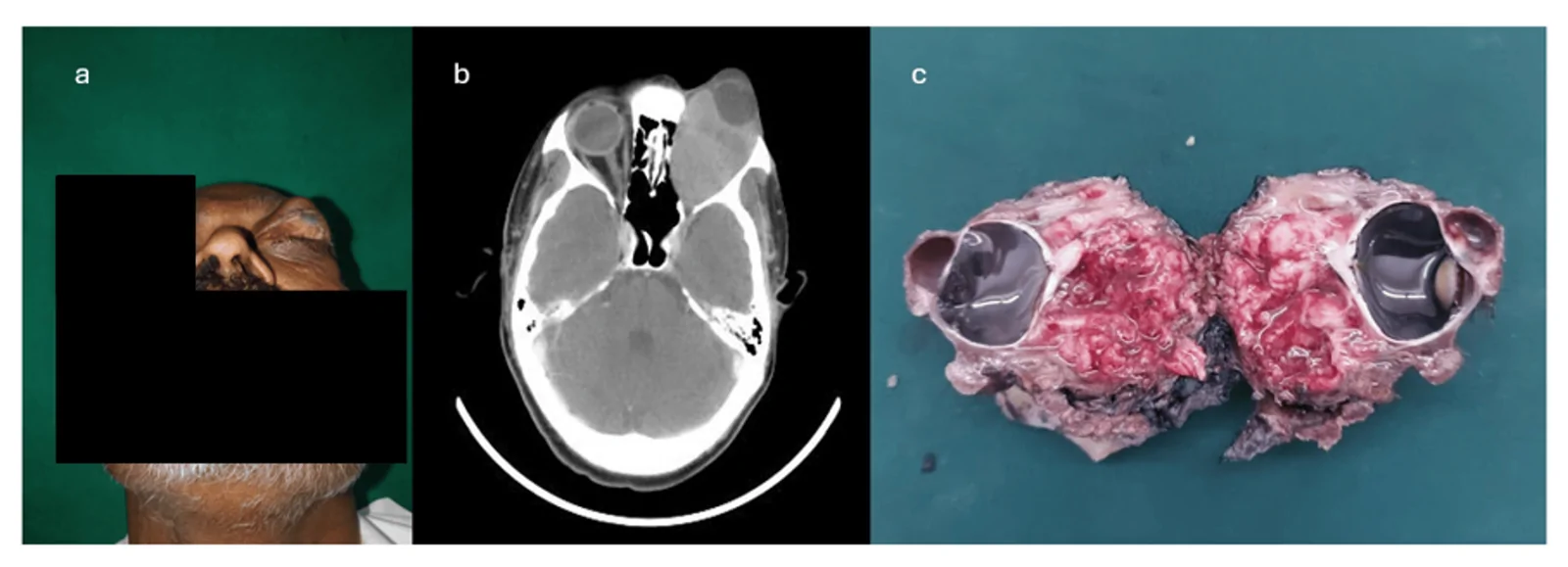
Clinical, radiological and gross photograph of orbital solitary fibrous tumor (SFT) of a 57-year-old male. Case 6 in Table 3: (a) Clinical image showing proptosis with lagophthalmos of left eye, (b) CT images showing a retrobulbar mass in the left orbit, and (c) Gross image showing a retrobulbar fleshy brown tumor with an uninvolved eyeball.

**Table 3:** Clinical, histological and immunohistochemical characteristics with risk assessment of cases of solitary fibrous tumor (SFT) from Demicco et al. HPF = high power field; ICH = immunohistochemistry; SOL = space-occupying lesion

Sr.No.	Age/Sex	Clinical symptoms	Size of the Tumor (cm)	Mitosis (per 10 HPF)	Necrosis (%)	Total score as per Demicco et al. [6]	Tumor Site - Diagnosis	IHC
1	53/F	Swelling in neck	6x5.5	0	0	1 (Low risk)	Left Supraclavicular region - Solitary Fibrous Tumor	Positive - Vimentin, CD 34, STAT 6, CD99
								Negative - CD31, SMA, Desmin, S100, BCL2
2	49/F	Mass in infraclavicular region	3.5x2.8	0	0	0 (Low risk)	Left Infraclavicular region - Solitary Fibrous Tumor	Positive - Vimentin, CD 34, STAT 6, BCL2
								Negative - CD31, SMA, Desmin, S100, CD99
3	29/F	Headache	6.8x4.4	2	0	2 (Low risk)	Right Temporal SOL - Solitary fibrous tumor (CNS WHO Grade 1)	Positive - Vimentin, CD 34, STAT 6, CD99, EMA (focal)
								Negative - SMA, CK, PR, Desmin, SMA, BCL2
4	60/F	Abdominal pain	7x6	0	0	2 (Low risk)	Pouch of Douglas - Solitary Fibrous Tumor	Positive - Vimentin, CD 34, STAT 6, CD99, BCL2 (focal)
								Negative - CD31, SMA, Desmin, S100, C-Kit
5	25/F	Mass in left thigh	3.9x3.7	0	0	0 (Low risk)	Left Thigh - Solitary Fibrous Tumor	Positive - Vimentin, CD 34, STAT 6, CD99, BCL2
								Negative - CD31, SMA, Desmin, S100
6	57/M	Lagophthalmos & proptosis	4x3.9	15	0	3 (Low risk)	Orbit - Solitary Fibrous Tumor with malignant transformation	Positive - Vimentin, CD 34, STAT 6, BCL2
								Negative – SMA, Desmin, EMA, CK, S100, CD99, MIB -20%
7	55/M	Growing mass in thigh	19x9	20	10	7 (High risk)	Posterior Thigh - Solitary Fibrous Tumor with malignant transformation to angiosarcoma	Positive - Vimentin, CD 34, STAT 6, BCL2, ERG, CK (focal), Desmin (Focal)
								Negative - SMA, S100, EMA, CD99, B catenin, Mib 1 – 40%
8	52/M	Mass in inguinal region	8x6	5	20	4 (Intermediate risk)	Right inguinal region - Solitary Fibrous Tumor	Positive - Vimentin, CD 34, STAT 6, CD99, BCL2
								Negative - CK, EMA, SMA, Desmin, S100, ALK, Mib 1 – 30%
9	75/M	Scrotal mass	8.7x3.6	10	20	5 (Intermediate risk)	Para-testicular region - Solitary Fibrous Tumor with malignant transformation	Positive - Vimentin, CD 34, STAT 6, CD99, BCL2, CD10 & B-Catenin
								Negative - CK, EMA, CD31, SMA, Desmin, S100, CD117, Calretinin MIB1- 60%
10	64/F	Chest pain, breathlessness	4.5x3	2	0	2 (Low risk)	Pleura	Positive - Vimentin, CD 34, STAT 6
								Negative - CD31, SMA, Desmin, S100, CD99
11	55/F	Chest pain & pleural mass	5.2x3.3	2	0	3 (Low risk)	Pleura	Positive - Vimentin, CD 34, STAT 6, CD99, BCL2
								Negative - CD31, SMA, Desmin, S100
12	58/M	Proptosis & diplopia	6x3	0	0	2 (Low risk)	Orbit	Positive - Vimentin, CD 34, STAT 6, BCL2
								Negative - CD31, SMA, Desmin, S100, CD99
13	46/M	Chest pain	6.3x3	2	0	2 (Low risk)	Pleura	Positive - Vimentin, CD 34, STAT 6, CD99
								Negative - CD31, SMA, Desmin, S100, CD99, BCL2, B- catenin
14	65/M	Chest pain & cough	5.5x4	0	0	2 (Low risk)	Left Pleura	Positive - Vimentin, CD 34, STAT 6
								Negative - CD31, SMA, Desmin, S100, CD99, BCL2
15	68/M	Breathlessness	5x4	0	0	2 (Low risk)	Lung and Mediastinum	Positive - Vimentin, CD 34, STAT 6, BCL2
								Negative - CD31, SMA, Desmin, S100, CD99
16	43/M	Chest pain, breathlessness	5x5	0	0	1 (Low risk)	Right Pleura	Positive - Vimentin, CD 34, STAT 6, CD99
								Negative - CD31, SMA, Desmin, S100, BCL2
17	40/F	10^th^ rib mass lesion	7.4x3.8 1	0	0	1 (Low risk)	Rib	Positive - Vimentin, CD34, STAT6, BCL2
								Negative - Desmin, SMA, S100, CD99, CD31
18	40/F	Swelling over right side of neck	5.2x4.5	0	0	1 (Low risk)	Right cervical swelling	Positive - Vimentin, CD34, STAT6, Desmin(Focal)
								Negative - CK, S100, SMA, Caldesmon
19	45/F	Right thoracic mass	11.4x 7.5	2-3	0	3 (Low risk)	Right pleural mass	Positive - Vimentin, STAT6, CD34
								Negative - CK, S100, SMA, Calretinin, TTF1
20	40/F	Lumbar mass	9.6x7.7	1-2	0	2 (Low risk)	Left lumbar region	Positive - Vimentin, CD34, STAT6, CD99
								Negative - Desmin, SMA, S100, CD31, BCL2

**Figure 4:**
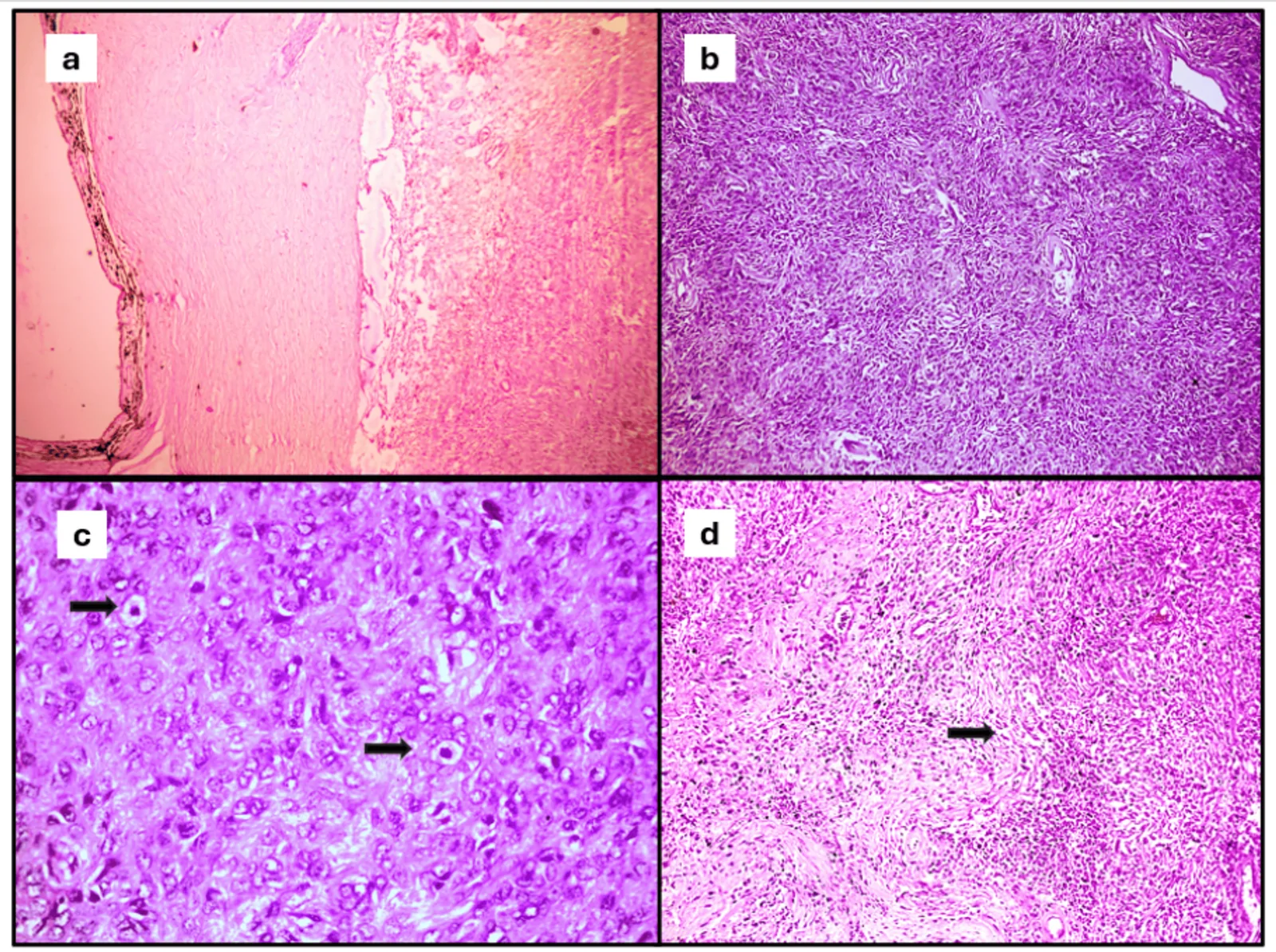
H&E images of orbital solitary fibrous tumor (SFT) of a 57-year-old male. Case 6 in Table 3: (a) A spindle cell tumor behind uninvolved eyeball (x40), (b) Areas exhibiting typical SFT morphology with a storiform pattern (x200), (c) Increased mitoses (arrows) (x400), (d) Foci of tumor necrosis (x100).

**Figure 5:**
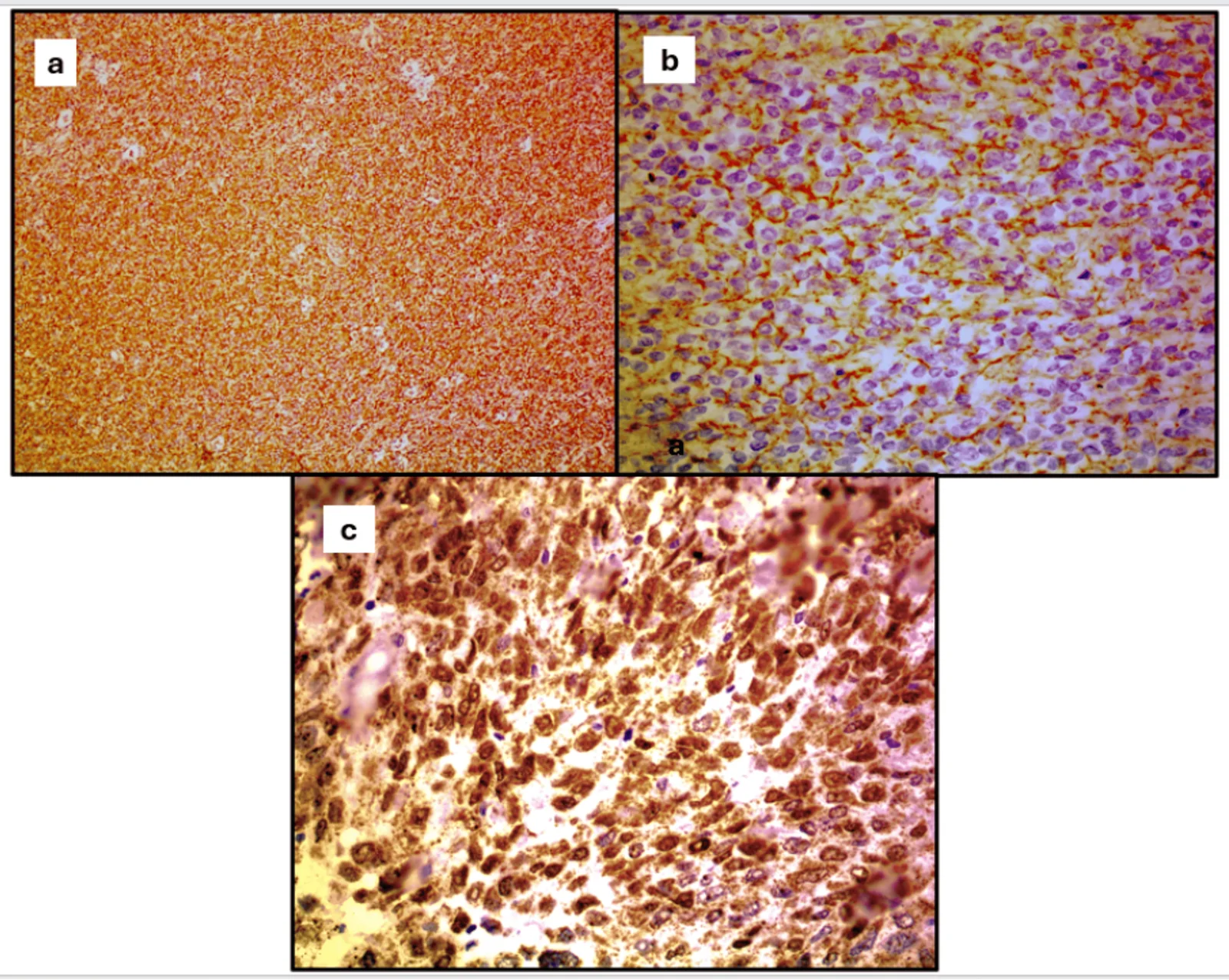
Orbital solitary fibrous tumor (SFT) immunohistochemistry images of a 57-year-old male. Case 6 in Table 3: Tumor cells expressing (a) CD34 (b) Vimentin, (c) showing bright nuclear expression of STAT6. (a),(b) and (c) x400.

One case showed angiosarcomatous transformation/dedifferentiation. The case was a 55-year-old male with a 19 cm mass in the posterior thigh. The H&E images showed a high-grade spindle cell tumor with areas with typical SFT-like morphology with a few areas showing vascular spaces lined by atypical cells, marked nuclear pleomorphism, areas of necrosis and brisk and atypical mitoses. IHC showed expression of vimentin, STAT6, ERG, and BCL2 with a Ki-67 labelling index of 40% and was negative for CD99, EMA, SMA and S100 (Figure 6).

**Figure 6:**
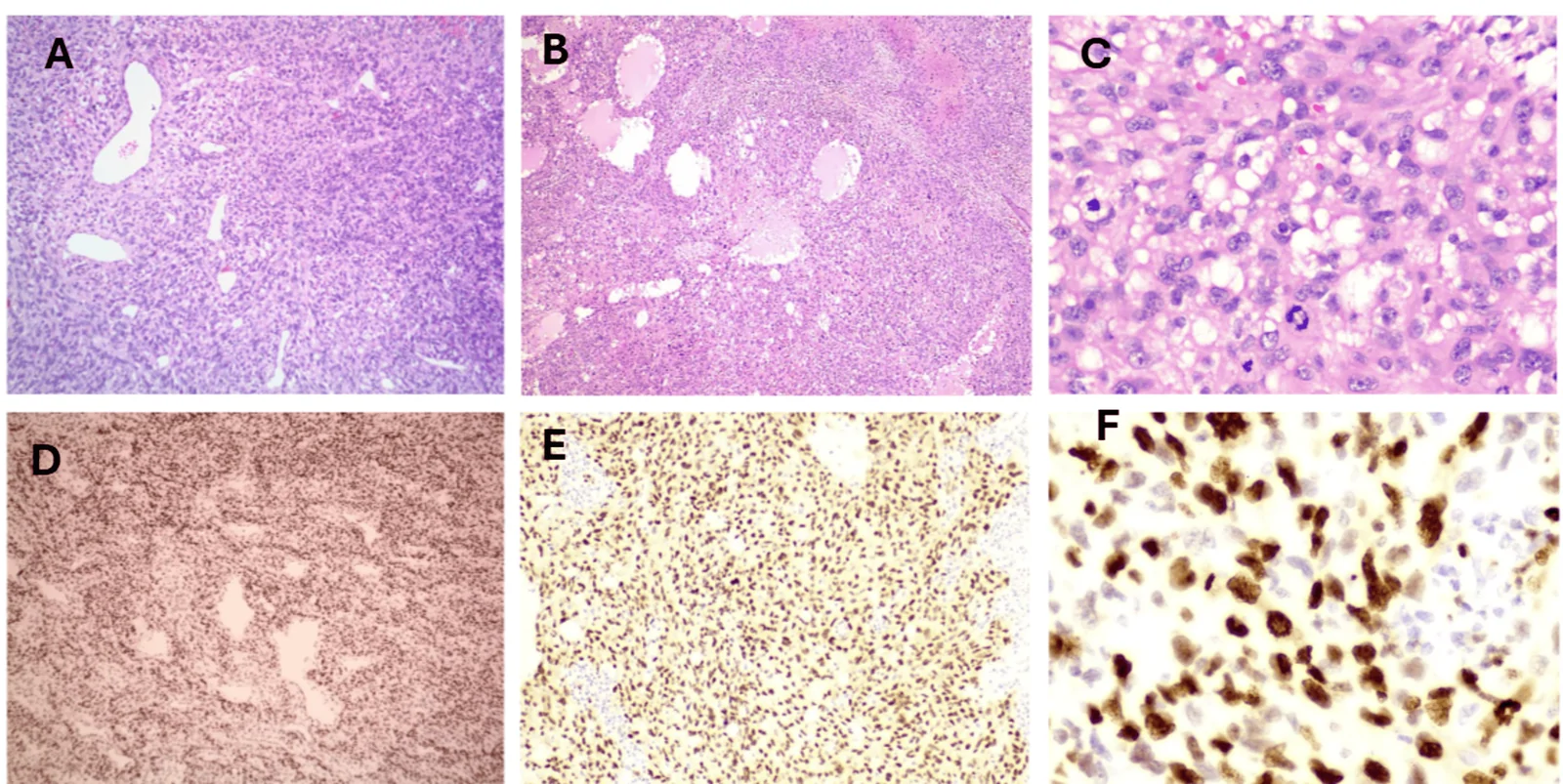
Solitary fibrous tumor (SFT) with angiosarcomatous de-differentiation (H&E and immunohistochemistry (IHC) images) of a 55-year-old male. Case 7 in Table 3: H&E images showing (a) cellular spindle to ovoid cells with hemangiopericytoma-like areas (x100) (b) Cellular tumor with areas of necrosis (x100) (c) epithelioid cells with marked nuclear pleomorphism and showing atypical mitotic figures. IHC images (d) Strong nuclear positivity for STAT6 (x100) (e) Strong nuclear positivity for ERG (x200) (f) Ki-67 labelling index around 40% (x400).

The cases were categorized based on risk assessment criteria like Demicco et al. [6] into low, intermediate and high risk (Table 3). Out of 20 cases one case was high risk, two were intermediate risk and the other 17 cases were in the low-risk category.

## Discussion

SFTs are rare mesenchymal neoplasms with a broad spectrum of anatomical distribution. Initially described in the pleura [7], these tumors have since been identified in various extra-thoracic locations. The most common sites include the pleura, soft tissues, and retroperitoneum, but they can also occur in more unusual sites such as the central nervous system, liver, orbit and paranasal sinuses [8].

In our study we found that 30% of cases arose in the pleura. This was comparable to studies done by Demicco et al. [1] and Devito et al. [2], who also found the incidence of pleural tumors as 28% and 34%, respectively. Our study also found SFT arising in very rare locations such as the orbit, temporal lobe of brain, lumbar region and paratesticular region. Our findings are comparable to the series by Angelico et al. [9], who analyzed 31 SFTs arising in unusual locations including parenchymal organs and deep soft tissues. Similar to our study, they emphasized the wide anatomical distribution, including sites such as the kidney, breast, and mesorectal tissue, reinforcing the concept that SFT can occur virtually anywhere.

The age distribution in our cohort was typically middle-aged to elderly adults, with a mean age of 50.9 years which was comparable to studies done by Demicco et al. and Friis et al. [1,7]. We had a slight female preponderance which is comparable to a study done by Devito et al. [2]. Demicco et al. [1] had an equal male-to-female ratio, while the study done by Friis et al. [7] showed a slight male preponderance.

According to a study done by Thway et al. [10], most of the cases presented with nonspecific symptoms like chest pain and mass which was comparable to our study. They also found that 75% of cases presented with symptoms after diagnosis, resulting in delay in diagnosing these tumors.

Histologically, the majority of cases in our study demonstrated classic features, including the “patternless pattern,” alternating hypercellular and hypocellular areas, and prominent branching (“staghorn”) vasculature. However, site-specific morphological variations were observed, particularly in small biopsies and deep-seated lesions, where increased cellularity and stromal hyalinization occasionally obscured the typical appearance. 

IHC proved indispensable in establishing the diagnosis. Diffuse nuclear expression of STAT6 was observed in the vast majority of our cases, supporting its role as a highly sensitive and specific marker [11]. Additional positivity for CD34 and BCL2 was noted in most tumors, although these markers lacked specificity. The identification of STAT6 nuclear staining, reflecting the underlying NAB2-STAT6 fusion, was particularly useful in resolving morphologically ambiguous cases and differentiating SFT from histological mimics such as spindle cell sarcomas, meningioma, and gastrointestinal stromal tumors.

In our study, three cases showed features associated with aggressive behavior, including increased mitotic activity (>4/10 HPF), nuclear atypia, and focal necrosis. These lesions include one case each involving orbit, paratesticular region and posterior thigh.

The malignant transformation in orbital SFT is very rare, accounting for 6-12% [12]. According to Demicco et al.'s risk stratification, this case falls in the low-risk category, thus misleading to the aggressiveness of the tumor [6]. According to Thompson et al. [13], who gave a risk assessment score for orbital SFT, our case falls in the intermediate risk category, thus proving to be a better model in the case of orbital SFT.

We had one case of malignant SFT from the para-testicular region which showed fibrosarcomatous transformation. There are fewer than 10 cases reported in the literature of SFT arising from the para-testicular region [14], thus making this case special.

The de-differentiation in SFT is very rare and generally the de-differentiated component is undifferentiated sarcoma, osteosarcoma or rhabdomyosarcoma [15]. In our case there was angiosarcomatous transformation. To our knowledge, there is very little literature available on angiosarcomatous transformation/de-differentiation in SFT and ours is the first case of the same.

Demicco et al. [1] developed a risk assessment model based on a large series of cases. This model incorporates tumor size, mitotic rate, and patient age to stratify patients into different risk categories for metastasis. They further modified the risk assessment in 2017 and added tumor necrosis to the scoring system. According to Demicco et al. [6], larger tumors, higher mitotic rates, and older age are associated with an increased risk of adverse outcomes​ [6]. Using these variables, three risk groups for recurrence are suggested: low (score 0-3), intermediate (score 4-5), and high risk (score 6-7). Risk stratification using Demicco et al. [6] revealed that a maximum proportion of tumors in our series fell into the low-risk category (17 cases). Only two cases were in the intermediate-risk category and one case in the high-risk category. Demicco et al. [6] also reported around 45% of cases in the low-risk category. With the Demicco et al. model we could identify two cases with malignant transformation. 

The present study has certain limitations. The small sample size of 20 cases reflects the rarity of SFTs and limits the statistical power and generalizability of the findings. The study being retrospective, it is associated with inherent selection bias and dependence on the completeness and accuracy of archived clinical and pathological records. The follow-up is limited, thus long-term outcomes such as local recurrence, metastasis, and disease-specific survival cannot be assessed. Molecular testing was not done due to non-availability; therefore, diagnosis relied primarily on characteristic histomorphological features and diffuse nuclear STAT6 immunohistochemical expression.

Thus, comparison with existing literature indicates that our findings are largely concordant with previously reported series, particularly regarding morphology, immunophenotype, and prognostic factors. However, the exclusive focus on unusual anatomical sites in our cohort adds to the growing body of evidence that SFT should be considered in the differential diagnosis of spindle cell tumors at any location.

## Conclusions

SFT is an uncommon mesenchymal neoplasm with a broad anatomical distribution and variable biological behavior. In our series of 20 cases arising from pleural and extrapleural sites, the tumors demonstrated considerable variation in size, cellularity, mitotic activity, and morphological features, highlighting the diagnostic and prognostic heterogeneity of SFTs. Our 20-case series reinforces the concept that SFT is a diagnostically challenging tumor in unusual locations.

Histopathological evaluation remains the cornerstone of diagnosis, while immunohistochemistry, particularly STAT6 nuclear expression, is important for establishing the diagnosis in morphologically challenging cases. The Demicco et al. risk assessment model, which incorporates patient age, tumor size, mitotic count, and necrosis, provides a useful and reproducible method for stratifying SFTs according to metastatic risk. In our series, application of this model demonstrated that tumors with unfavorable clinicopathological parameters tended to fall into higher-risk categories, supporting the applicability of the model across different anatomical sites.

Larger multicentric studies with long-term clinical follow-up are required to further validate risk stratification models and determine their predictive performance across diverse anatomical sites. Integration of morphology, STAT6-based immunohistochemistry, and validated risk assessment systems can provide a more comprehensive approach to diagnosis, prognostication, and clinical management of patients with SFT.
